# Tactile-Sensing Based on Flexible PVDF Nanofibers via Electrospinning: A Review

**DOI:** 10.3390/s18020330

**Published:** 2018-01-24

**Authors:** Xiaomei Wang, Fazhe Sun, Guangchao Yin, Yuting Wang, Bo Liu, Mingdong Dong

**Affiliations:** 1 Laboratory of Functional Molecules and Materials, School of Physics and Optoelectronic Engineering, Shandong University of Technology, Zibo 255049, China; xiaomeiwang@sdut.edu.cn (X.W.); yingc@sdut.edu.cn (G.Y.); 2 Analysis Testing Center, Shandong University of Technology, Zibo 255100, China; sunfazhe@163.com; 3 Interdisciplinary Nanoscience Center (iNANO), Aarhus University, DK-8000 Aarhus C, Denmark; wangyuting949@163.com

**Keywords:** PVDF, P(VDF-TrFE), electrospinning, flexible tactile sensor

## Abstract

The flexible tactile sensor has attracted widespread attention because of its great flexibility, high sensitivity, and large workable range. It can be integrated into clothing, electronic skin, or mounted on to human skin. Various nanostructured materials and nanocomposites with high flexibility and electrical performance have been widely utilized as functional materials in flexible tactile sensors. Polymer nanomaterials, representing the most promising materials, especially polyvinylidene fluoride (PVDF), PVDF co-polymer and their nanocomposites with ultra-sensitivity, high deformability, outstanding chemical resistance, high thermal stability and low permittivity, can meet the flexibility requirements for dynamic tactile sensing in wearable electronics. Electrospinning has been recognized as an excellent straightforward and versatile technique for preparing nanofiber materials. This review will present a brief overview of the recent advances in PVDF nanofibers by electrospinning for flexible tactile sensor applications. PVDF, PVDF co-polymers and their nanocomposites have been successfully formed as ultrafine nanofibers, even as randomly oriented PVDF nanofibers by electrospinning. These nanofibers used as the functional layers in flexible tactile sensors have been reviewed briefly in this paper. The β-phase content, which is the strongest polar moment contributing to piezoelectric properties among all the crystalline phases of PVDF, can be improved by adjusting the technical parameters in electrospun PVDF process. The piezoelectric properties and the sensibility for the pressure sensor are improved greatly when the PVDF fibers become more oriented. The tactile performance of PVDF composite nanofibers can be further promoted by doping with nanofillers and nanoclay. Electrospun P(VDF-TrFE) nanofiber mats used for the 3D pressure sensor achieved excellent sensitivity, even at 0.1 Pa. The most significant enhancement is that the aligned electrospun core-shell P(VDF-TrFE) nanofibers exhibited almost 40 times higher sensitivity than that of pressure sensor based on thin-film PVDF.

## 1. Introduction

With the explosive development of science and technology, widespread attention has been paid to tactile sensors on both the research and applied technology fields [1,2]. Tactile sensing provides measurable information by given properties of an object or event through a tactile sensor. These are responsive sensors that are defined as detecting and measuring electrical parameters to monitor human activities, physiological signals, and analyzing these signals when given physical contact such as mechanical deformation [3,4]. The tactile sensor are classified by a few groups such as piezoelectric, piezoresistive, triboelectric, capacitive, and optical sensors according to the sensing mechanism employed. A good tactile sensor should be highly sensitive, stable, simple, and quickly responsive [5]. However, due to the limitation of the mechanical properties (including stretching, bending, pressure and torsion) [6,7], the development of the traditional piezoelectric sensors were greatly hindered [8,9,10]. The piezoelectric sensors can be classified into two groups: ceramic and polymeric ones. There are some disadvantages for piezoelectric ceramic based on inorganic materials such as high temperature for polar reorientation, high cost preparation process, and poor flexibility. Piezoelectric polymeric materials, on the other hand, have excellent mechanical flexibility, multi-technology compatibility, and cheaper cost. The most prominent advantage is that it has good nanomaterial formability. Therefore, the use of piezoelectric polymeric materials in the tactile sensor has been developing rapidly. Meanwhile, various nanostructured materials (nanofibers/nanowires, graphene and carbon nanotubes, polymer nanofibers, etc.) [11,12,13,14,15,16,17,18] and nanocomposites [19,20,21,22] with high flexibility and electrical performance have been widely utilized as functional materials and the rigid substrates have been replaced by flexible substrates including polyethylene terephthalate, polyurethane, and polydimethylsiloxane (PDMS), which has promoted the development of flexible tactile sensors. Rapid development has been achieved in recent years to fabricate flexible tactile sensors, due to the ultrahigh stretchable, bending, and wearable features, which can be easily integrated into artificial and electronic skin (e-skin) [23,24,25,26]. 

Polymer nanomaterials, especially polyvinylidene fluoride (PVDF), PVDF co-polymers and their nanocomposites with ultra-sensitivity and high deformability, represent the most promising material, which can meet the hot spot of flexibility and the requirements of dynamic tactile sensing in wearable electronics [27,28,29]. Various fabrication methods of nanomaterials such as sol-gel [30], chemical vapor deposition (CVD) [2,6], hydrothermal method [31], etc., are adopted, but are generally limited by complicated processes, high-temperature treatment, high-vacuum system or high cost. Electrospinning is a typical one-step process, low-cost, and versatile technique to fabricate nanofibers from solution at room temperature, which has been widely utilized to fabricate one-dimensional polymer nanofibers [32]. The crystallinity of a polymer nanofiber can be significantly improved due to the ultra-high specific surface area and aspect ratio and therefore the electrical performance can be further enhanced [28] by electrospinning. 

The nanofiber materials of PVDF, PVDF co-polymers and their nanocomposites, even randomly oriented PVDF nanofibers, have been successfully fabricated [33] and used for the functional layer of flexible tactile sensors with excellent performance. This review will present a brief overview of the recent advances in PVDF nanofibers by electrospinning for flexible tactile sensor applications. As the functional materials of the tactile sensors, PVDF nanofibers by electrospinning are mainly used for piezoelectric sensors and piezoresistive sensor. Most PVDF nanofiber-based tactile sensors have a common mechanism working by piezoelectricity. The β-phase content contributing to piezoelectric properties can be improved by adjusting the technical parameters during the electrospun PVDF process. The tactile performance of the PVDF composite nanofibers doped with nanofillers and nanoclay used in sensors can be further promoted. Electrospun P(VDF-TrFE) nanofiber mats achieved excellent sensitivity at exceptionally low pressure (0.1 Pa) [34]. In addition, the excellent sensitivity can even be applied to 3D pressure sensor [35]. The piezoelectric properties are greatly improved when the arrangement of PVDF fibers become more oriented. The most significant enhancement is that the aligned electrospun P(VDF-TrFE) core–shell nanofibers were fabricated and exhibited almost 40 times higher sensitivity than that of a pressure sensor based on thin-film PVDF. Both PVDF nanocomposite (such as PVDF/Ag and PVDF/PPy) and P(VDF-TrFE) nanofibers by electrospinning show excellent pressure-sensor performance while working by the mechanism of piezoresistivity.

## 2. Electrospinning

Electrospinning is a simple, low-cost, and versatile technique to fabricate long and continuous micro/nanoscale fibers (from a few nanometers to submicrometers) [36,37]. The invention of electrospinning was patented in 1934 [38]. Thereafter, this technology has attracted growing industrial and academic attention [32,39,40].

**Figure 1:**
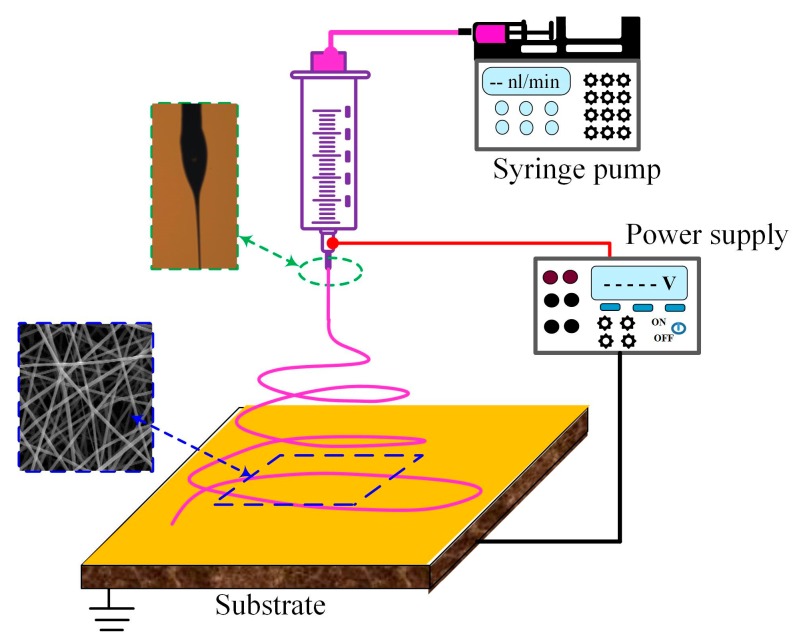
Schematic illustration of an electrospinning.

The schematic illustration of a laboratory electrospinning setup is shown in Figure 1. A high positive voltage is applied to a polymer fluid or melts through a needle with respect to the substrate or nanofiber collector. The applied voltage produces a high electrical field, and when the electrical field force of the accumulated ions in the solution at the air-fluid interface overcome the surface tension, a fluid jet erupts. The jet can achieve a strong reduced diameter to nanoscale while traveling during the flight [41]. The jet between the needle tip and the collector is divided into three regions [42]: (i) a droplet region of conical shape called Taylor cone at the tip of the needle due to the balance between electrostatic force and surface tension [43]; (ii) a stable straight jet region after Taylor cone region and (iii) an unstable whipping motion region with the lateral coils formed by jet running and a cone-shape envelope opening towards the collector/substrate [44]. Many kinds of nanofibers with different morphologies can be achieved when fibers are collected at different regions of the jets. The region (iii) has three unstable whipping stages [41], so the disorganized fibers are collected as a nonwoven mat. When the fibers are collected at a stable straight region (ii), well-aligned nanofibers and orderly nanofiber patterns can be obtained [45]. When the straight region is less than 1 mm and 1–10 mm with mechanical movement of the collector, the electrospun processes are called near-field electrospinning [46] and mechanoelectrospinning [47], respectively. In addition, core-shell, hollow or tied fibers can be fabricated by using coaxial or bicomponent spinneret during electrospinning [39].

The morphologies of nanofibers by electrospinning also can be influenced by multiple factors [48] including: (a) the solution parameters such as viscosity, surface tension, dielectric constant, and conductivity, (b) process variables such as voltage and distance between the needle tip, and the collector, flow rate of fluid; and (c) ambient parameters including temperature, humidity, and air velocity in the electrospinning chamber [49]. Two common strategies have been proposed to optimize the orientation of the nanofibers [39] by electrospinning. One strategy is introducing the parallel electrode collector where the fibers can be stretched perpendicular to the edges of the gap due to the traction of electrostatic forces. Meanwhile, the fibers’ alignment can be significantly improved by decreasing the distance between the parallel electrodes [50]. However, the above fiber layers suffer partial misalignment [51]. Another strategy is introducing rotating collector [39] where the fibers can stretch along the rotating drum due to the traction force from the rotating speed. However, the relation between the linear speed of the dynamic collector surface and the jet deposition is a critical factor by electrospinning. The tip-to-collector distance is also identified as the important relevant factor [52] for an effective control on fibers’ alignment. The jet can also be controlled by applying magnetic fields [53] to polymer solutions containing magnetic nanoparticles. Employing auxiliary electrodes [54], modifying the electrical field, or an electrically earthed collector [51] to collect the nanofibers can also enhance the alignment of nanofibers.

Among the electrospun piezoelectric materials applied in tactile sensors, electroactive piezopolymers such as polyvinylidene fluoride (PVDF) (*d*_31_≈20 pC/N and −20 ≥ *d*_33_ ≥ −30 pC/N) [55] and its co-polymer polyvinylidene fluoride-trifluoroethylene (P(VDF-TrFE)) (*d*_31_ ≈ 25 pC/N and −30 ≥ *d*_33_ ≥ −40 pC/N) [56] have been extensively researched in flexible piezoelectric applications owing to their significant advantages, such as excellent piezoelectric performances, flexibility, breathable properties, and long-term stability. Moreover, the PVDF and P(VDF-TrFE) mats are applicable to curved surfaces and work in the dynamic environment for tactile applications.

## 3. Polyvinylidene Fluoride Piezoelectric Material

### 3.1. Piezoelectric Polyvinylidene Fluoride

**Figure 2:**
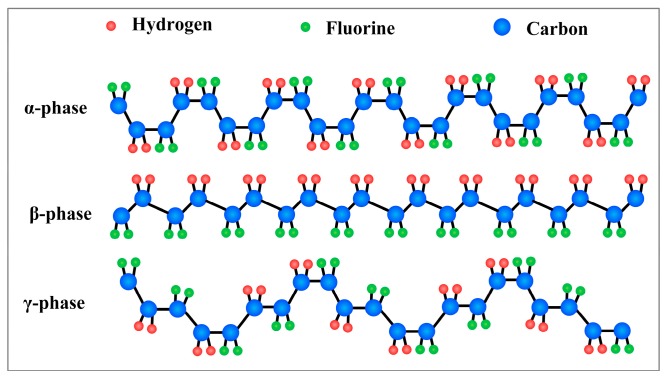
Schematic representation of the chain conformation for the α, β and γ-phases of polyvinylidene fluoride (PVDF).

PVDF, whose molecular formula is (–CH_2_–CF_2_–)*_n_*, is a semicrystalline homopolymer, and the crystallite polymorphs have five phases (α, β, γ, δ, and ε phases) owing to fabricating conditions [57]. The range of electrical dipole moment of the PVDF monomer unit for different phases is 5–8 × 10^−30^ C·m [58,59]. Figure 2 shows the schematic representation of the chain conformation for the most investigated PVDF phases: α, γ and β-phases. Piezoelectricity is the electric polarization of electric dipole moments in anisotropic crystalline materials while applying mechanical stimulation [60]. So the piezoelectric properties of PVDF are mainly depending on the polar crystalline phases including β-phase (TTTT conformation) and γ-phase (TTTGTTTG′ conformation), but not including α-phase which has a nonpolar TGTG′ conformation. The β-phase is the strongest polar moment and most electrically active phase among these crystalline phases, which shows the highest electrical dipole moment(8 × 10^−30^ C·m) [61]. The polarity of the γ-phase is between that of the α- and β-phases. Thus there is great significance to increase the content of β-phase in PVDF material for the sensor performance [62]. Many methods are researched to enhance the content of β-phase in PVDF.

Various efficient techniques have been applied during the fabrication process to achieve dipole alignment of the crystalline structures in β-phase PVDF, such as uniaxial or biaxial stretching [63,64,65], thermal annealing [62,66], high electrical field [67], surface charge [68,69], and fillers [70,71,72]. Multiple nano-fillers acting as nucleating agents, such as graphene [73,74], carbon nanotubes (CNT) [75,76,77,78,79] including single-wall carbon nanotube (SWCNT) and multi-wall carbon nanotube (MWCNT), and clay [80,81], have been added to enhance the formation of β-phase. It is reported an increased crystallization of β-phase resulted when the nano-fillers content increased [75,82]. 

### 3.2. Electrospun Polyvinylidene Fluoride

The nanoscale fibers of α or β or γ-phase PVDF with an increasing content of β-phase could be fabricated directly by electrospinning without any post-treatment. Zheng et al. [83] found that the β-phase content can be improved by adjusting the technical parameters during electrospinning, such as adding a low boiling point solvent (acetone), decreasing the environment temperature, or decreasing the flow rate and distance between the tip and the substrate/collector. The high stretching ratio of the jets, due to the high voltage applied to the precursor solution, is beneficial to the formation of β-phase PVDF [32], and the high ratio of stretching by electrospinning is similar to uniaxial mechanical stretching, which makes the phase transition from α-phase to β-phase [63,65,84]. Both the low environmental temperature at the room temperature range (20–60 °C), which provides the low crystallization temperature for the jets, and the rapid evaporation of the solvent are also beneficial to the formation of β-phase PVDF [83].

**Figure 3:**
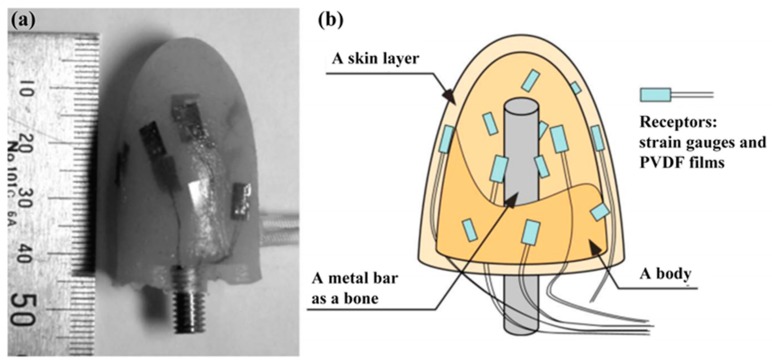
A developed fingertip (a) and its cross sectional sketch (b) [87].

The flexible PVDF material used as a tactile sensor shows several advantages, such as high piezoelectric coefficient, simple processing technology, dimensional stability, and chemical inertness [85]. The typical frequency range is between 10 Hz and 50 kHz [86] for the well-known dynamic measurement method to achieve high sensitivity and transient sensing capabilities. An anthropomorphic soft fingertip designed by Koh Hosoda et al. constituted of a metal bar as the bone, a body, and a skin layer of silicon rubber, whose structure is similar to a human finger (Figure 3). PVDF-based sensors were mounted on the fingertip by randomly embedded in the body of the soft fingertip and the skin layer as receptors [87], and can discriminate five different materials to collect texture information by pushing and rubbing the objects. Based on PVDF nanofibrous fabrics, preliminary force sensors with excellent flexibility and breathability have been fabricated by electrospinning and demonstrated superior response and sensitivity under external mechanical forces [28]. With the development of electrospinning device, aligned poly (vinylidene fluoride) (a-PVDF) fibers have been prepared [33,88,89], which can be used as functional layers in tactile sensors with excellent performance. Dezhi Wu et al. [18] prepared air pressure sensors based on a-PVDF fibers with 440~475 nm diameters on the soft substrate of polyethylene terephthalate (PET) and PDMS. The electrical field was modified by a parallel top-bottom collector configured with two kinds of materials during the non-uniform field electrospinning. The orientation of PVDF fibers became more aligned and the piezoelectric performances were greatly enhanced by the increasing thickness of PETs. The sensibility of the pressure sensor based on PVDF nanofibers was 5.812 mV kPa^−1^ and the output voltage signal was about 100 mV at 0.025 MPa air pressure. However, the PVDF material shows a susceptibility to temperature [90,91], especially temperature gradients. A cross-talk from temperature variation is undesirable [60]. So protection from thermal interference is necessary for PVDF used as piezoelectric materials.

### 3.3. Polyvinylidene Fluoride Nanocomposite

PVDF composites with nanostructure materials can promote better performance [70,92,93], thus the piezoelectricity of PVDF complex nanofibers used in a tactile sensor can be improved by doping with nanofillers and nanoclay. Multi-walled carbon nanotubes (MWCNTs) are commonly used as nanofillers in the preparation of PVDF complex by electrospinning. The β-phase composition and output signal for PVDF nanofibers were improved by the addition of MWCNTs compared with bare PVDF nanofibers [94]. The enhancement of MWCNT on a single PVDF nanofiber can also be proven by the increasing crystallinity of the piezoelectric β-phase crystal and the enhancing piezoelectric properties [94]. PVDF nanocomposites doped with MWCNT and cloisite 30B (OMMT) nanoclay were prepared by electrospinning [93]. It was observed that OMMT increases β-phase crystals and piezoelectric properties of PVDF as compared with MWCNT, and there was no synergistic effect of OMMT and MWCNT. The α-phase could be completely removed during the preparation of PVDF/OMMT composite nanofibers by electrospinning [95]. The uniformly dispersed OMMT precursor in PVDF can achieve intercalated and exfoliated, and the obtained PVDF/OMMT composite nanofibers were thinner than the pure PVDF nanofibers. The relaxation of PVDF chains can be retarded by OMMT platelets. Meanwhile, long trans conformation can be formed and stabilized owing to the interaction between OMMT filler and PVDF matrix during electrospinning. Such a cooperative effect leads to an extinction of α-phase and an increase of polar β and γ-phases in electrospun PVDF/OMMT composite nanofibers.

Baozhang Li et al. [96] prepared PVDF/Ag nanowire (AgNWs) composite nanofibers by doping AgNWs into PVDF via electrospinning. The results revealed that the content of β-phase in PVDF increased by adding AgNW and the piezoelectricity of PVDF nanofibers were greatly enhanced due to the interactions between the AgNWs and the PVDF matrix, which forces PVDF chains to form β phases [97]. The β phase and the sensitivity was found to be enhanced while increasing the content of AgNW. The sensitivity of PVDF/AgNWs (1.5 wt %) nanocomposite nanofibers used for pressure sensors can achieve 30 pC/N. 

The electrospun PPy particle-dispersed nanofiber webs exhibited a high surface area and a presence of piezoelectric β-phase [98]. Conductive PVDF/PPy nanofibers were fabricated using pyrrole (Py) oxidative polymerization on electrospun PVDF mats, which are suitably applied for pressure sensors [99]. PPy as a conducting polymer layer completely coated on the surface of PVDF fibers, and the relative conductivity was improved significantly under compressive stress while the Py concentration increased. The improvement of the PPy layer on electrical conductivity was attributed to two explained mechanisms: (1) A network structure of PVDF/PPy with randomly oriented fibers are forced to touch each other under pressure, reducing the distance and increasing the contact between PVDF/PPy fibers, which is beneficial to form new conducting pathways during compressive stress [98,99]; (2) The charge transport in PPy shows strong dependence on PPy chain orientation. The PPy chains were induced to polymerize in a preferential direction (along the fiber axis) by electrospinning [100], which strongly enhances the electrical conductivity of the samples [101]. The maximum electrical sensitivity of composite nanofibers was found when the PPy content is 50 wt %, and the relative conductivity increases about 40 times with applied compressive stress. Moreover, the electrical conductivity is almost unchanged as its previous value after the loading was released. Meanwhile, the conductivity of the PPy layer increases with conjugation, which is critically dependent on the synthesis time [101]. The electrical resistivity response of the PVDF/PPy blends is highly reproducible after repeated loading-unloading cycles [98]. PVDF/PPy blends fabricated with 13 wt % PPy particles displayed the highest sensitivity, with a change in electrical resistivity of about 10 orders of magnitude (from 10^17^ to 10^7^ Ω·cm) under 5 MPa compressive stress. The obtained non-woven mats with PVDF/PPy blends can be used in a pressure sensor.

## 4. Polyvinylidene Fluoride Co-Polymer: Poly(vinylidene fluoride trifluoroethylene)

**Figure 4:**
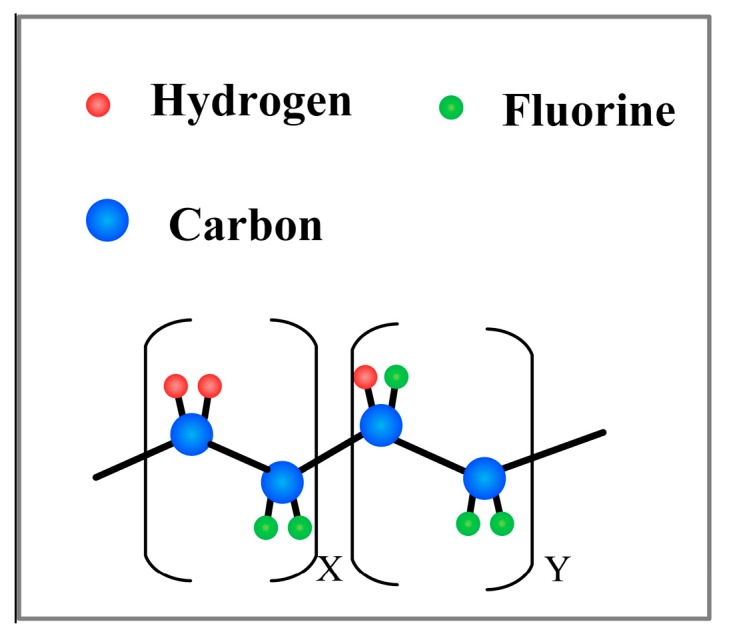
Schematic representation of the Poly(vinylidene fluoride trifluoroethylene) (P(VDF-TrFE)) repeat units.

The PVDF co-polymer such as Poly (vinylidene fluoride-hexafluoropropylene) (PVDF-HFP), Poly(vinylidene fluoride trifluoroethylene) (P(VDF-TrFE)) [102], P(VDF-TrFE) (Figure 4) is a kind of ferroelectric material with excellent piezoelectric effect, chemical stability and biocompatibility. Recently, P(VDF-TrFE) nanofiber mats have attracted increasing attention because of its flexibility, light weight, and low cost as a functional material for flexible tactile sensors. It is feasible to obtain a better piezoelectric response than PVDF thin films [103], as well as large effective working areas [104] and highly stretchable structures [105]. Moreover, the β-phase of the P(VDF-TrFE) indicate the crystallization preferentially and high piezoelectricity [106,107]. Even if PVDF generally presents a higher degree of crystallinity [84], the TrFE content facilitates the crystallization in the polymer chain, resulting in the higher content of the β-phase [107,108]. Further, the spherulite size and organization of polymers and copolymers on the surface morphology are generally different [109]. Meanwhile, the P(VDF-TrFE) flexible piezoelectric fibers achieved aligned arrangements, in which the polymer chains exhibited strong preferential orientation [34] and the piezoelectric performance were enhanced. Therefore, the electrospun P(VDF-TrFE) mats were used as the core piezoelectric layer in the tactile sensor.

**Figure 5:**
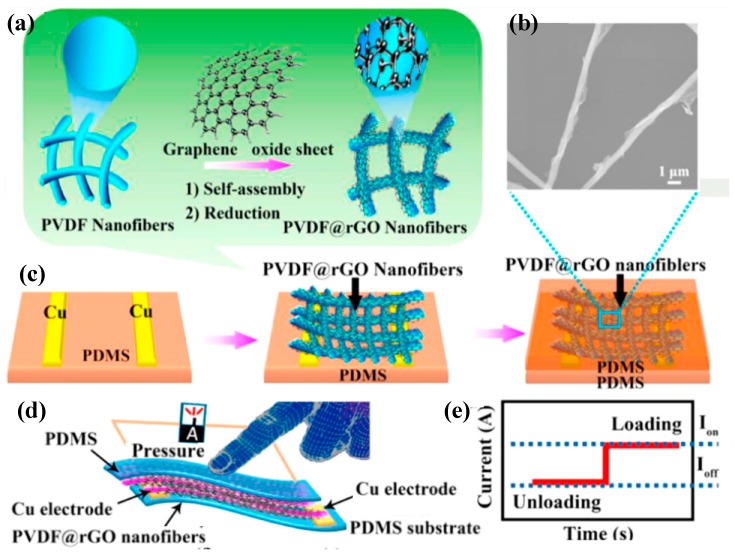
(**a**) Schematic illustration of the mechanism for the formation of PVDF fibers coated by the rGO nanosheets, followed by electrostatic interaction. (**b**) Field emission scanning electron microscopy (FESEM) image of the morphology of rGO nanosheets coated PVDF fibers. (**c**) Schematic illustration of the fabrication of a flexible pressure sensor. (**d**) Schematic of a typical pressure sensor. (**e**) Current changes in responses to loading and unloading [114].

The preferential orientation of CF_2_ dipoles in the P(VDF-TrFE) nanofiber, which was enhanced by the electrospinning process [110], has been investigated by Dipankar Mandal et al. The electrospun P(VDF-TrFE) nanofibers used for both piezoelectric and friction functional layers were fabricated on flexible films, which worked under piezoelectric and triboelectric hybrid stimulations [111]. The output voltage, power and power density of such product are respectively 25 V, 98.56 µW and 1.98 mW·cm^−3^ under triboelectric mechanism and 5 N pressure force. A maximum sensitivity of 60.5 mV·N^−1^ for electrospun P(VDF-TrFE; 77/23) nanofibers has been realized, which could be a reliably measured dynamic force up to a frequency of 20 Hz [112]. The obtained nanofiber mats can be used for tactile-sensor devices in practical applications because of their optimized structure and output performances. A highly flexible piezoelectric pressure sensor based on electrospun P(VDF-TrFE) nanofibers [113] was developed for measuring muscle movement on human skin. The orientation and polarization direction of P(VDF-TrFE) nanofibers were studied to improve the sensitivity of the pressure sensor, which can reach up to 110.37 pC·Pa^−1^ with a high signal to noise ratio. An ultra-sensitivity pizeoresistive pressure sensor based on 3D layers of the graphene oxide (rGO)-encapsulated P(VDF-TrFE) nanofibers (Figure 5)was successfully fabricated for the first time for applications as wearable electronics and electronic skins [114]. The sensor displayed excellent sensitivity (15.6 kPa^−1^), low detection limit (1.2 Pa), rapid response speed (5 ms), low operating voltage (1 V) at 50 Hz and long-term stability under 100,000 cycles. A super flexible strain sensor based on the nanocomposites of (P(VDF-TrFE)) fibers and ZnO nanowires has been fabricated on PDMS substrate [26]. The sensor exhibits a high sensitivity and rapid response (≈0.4 s), withstanding an ultimate stretch ratio up to 30%, and outstanding properties with a bending angle that changed up to 150°. 

**Figure 6:**
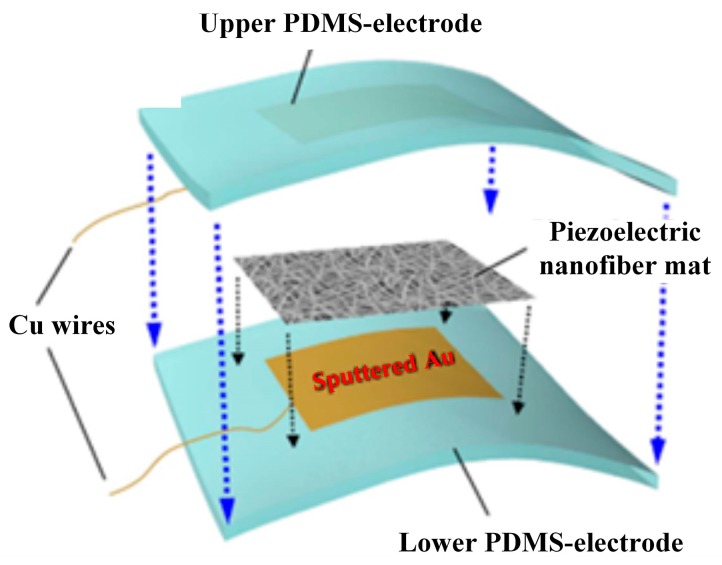
Schematic illustration of the configuration of the three-dimensional (3D) flexible piezoelectric sensor [35].

Electrospun P(VDF-TrFE) nanofiber mats used for 3D sensors including non-bent flat sensors, bent state sensors and finger-shaped sensors have been reported by Han Bit Lee et al. [35]. A stable linear relationship of input-output signals have been provided. The piezoelectric performance of normal *d*_33_ mode has been obtained for non-bent flat sensors, since the dipoles were mainly formed in the vertical direction. The output signals decrease slightly in bent state sensors due to the directions of the dipoles deviating along the direction perpendicular to the bent curve. The further lowered output data was received for finger-like sensors owing to their smaller size and the reduced surface area of the piezoelectric layer. Such 3D flexible customizable sensors (Figure 6), considered for application as wearable electronics, can provide high sensitivity to microscale deformation or force and stable linearity of the input-output relationship, and have greater functionality and suitability than traditional flat sensors in wearable and flexible electronic devices.

**Figure 7:**
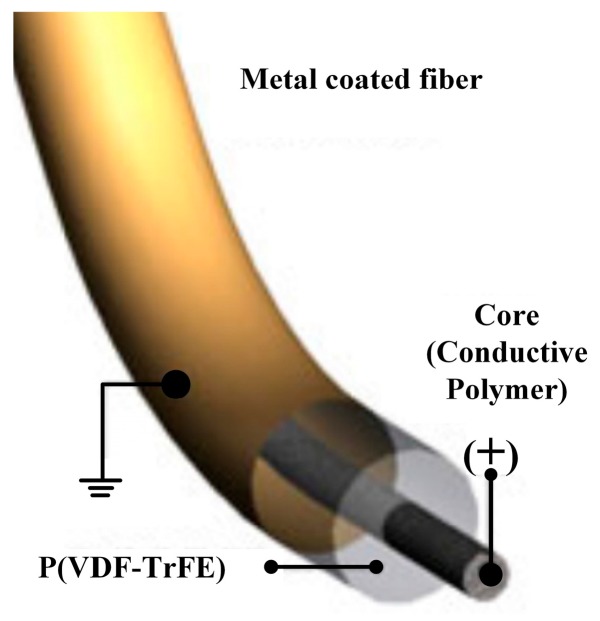
The sandwiched structure of piezoelectric polymer layers in core–shell fiber [116].

With the rapid development of electrospinning technology, the highly aligned nanofibers of P (VDF-TrFE) have been successfully fabricated on flexible substrates by one step and have been used for pressure sensors [115]. The tested results show that the device could produce 300 mV signals with a recovery time of 0.45 s on manual mechanical deformation. A large area aligned P(VDF-TrFE) fibers [34] for flexible piezoelectric material can be fabricated on free-standing and three-dimensional (3D) sheets by the electrospinning process associated with a fast rotating collector. The pressure sensor based on such fibers shows excellent sensitivity, even at 0.1 Pa. A more significant enhancement of aligned electrospun P(VDF-TrFE) nanofibers was that the core-shell nanostructures have been achieved [116]. The pressure sensor based on P(VDF-TrFE) (shell)-PVP/PEDOT: PSS (core) nanofibers (Figure 7) exhibited 4.5 times higher sensitivity than the aligned nanofiber-based devices and almost 40 times higher sensitivity than that of pressure sensors based on thin-film PVDF under in vitro simulated physiological conditions. However, just one single paper about the enhancement of the aligned electrospun P(VDF-TrFE) core-shell nanofibers on pressure sensing performance was published. 

The tested piezoelectric properties of the PVDF, P(VDF-TrFE) and their nanocomposite nanofibers or mats via electrospinning used for flexible tactile-sensing application have been summed up in Table 1. The output voltage of the tactile sensor based on PVDF nanofibers is of the order of 1 V (0.1–2.6 V). The output voltage and the sensitivity of PVDF nanofibers could increase by composited with nanofillers such as Ag nanowire, MWCNT, etc., because the nanofillers enhance the content of crystalline polar β-phase in composites. The relative conductivity and the sensitivity of pressure sensor were improved significantly while PVDF nanofibers doped with PPy. Compared with PVDF nanofibers, the piezoelectric output voltage of P(VDF-TrFE) nanofibers have no obvious increase. However, the triboelectric voltage of P(VDF-TrFE) nanofibers (25 V) is much higher than the piezoelectric voltage (2.5 V). Furthermore, P(VDF-TrFE) nanofibers have better sensitivity, lower detection limit and better stability than PVDF nanofibers. The output piezoelectric voltage of 3D sensor based on P(VDF-TrFE) nanofibers is lower than that of traditional 2D sensor based on P(VDF-TrFE) nanofibers, but the sensitivity of 3D sensor is much larger than that of the latter. In addition, the output voltage of P(VDF-TrFE) (shell)-PVP/PEDOT: PSS (core) nanofibers working by piezoelectricity is slightly higher than that of P(VDF-TrFE) nanofibers. So P(VDF-TrFE) based nanofiber materials are considered as ideal functional materials for the flexible tactile sensor.

## 5. Conclusions

PVDF nanostructure materials with ultra-sensitivity, high deformability, outstanding chemical resistance, high thermal stability, low permitivities and low acoustic impedances can meet the requirement of flexibility for dynamic tactile sensing in wearable electronics. The electrospinning technique becomes available to fabricate PVDF nanofiber layers with excellent performance for flexible tactile-sensing applications in a one-step, low-cost and versatile style. Moreover, the β-phase content and piezoelectric properties of PVDF can be improved by adjusting the electrospinning technical parameters. It becomes exceedingly essential to design different morphologies of nanofibers or mats during electrospinning in order to properly enhance the performances of tactile sensors. Now the main tactile function layer in 3D pressure sensors is mainly composed by electrospun PVDF and PVDF co-polymer nanofibers mats, which can achieve excellent sensitivity. The tactile performances of PVDF are greatly improved when PVDF and PVDF nanofibers become more oriented or doped with nanofillers and nanoclay. The most significant enhancement is that the aligned electrospun P(VDF-TrFE) core–shell nanofibers were successfully fabricated, and the core–shell nanofibers exhibit almost 40 times higher sensitivity than that of pressure sensors based on thin-film PVDF. Both PVDF nanocomposite and co-polymer nanofibers by electrospinning exhibit excellent piezoresistive performance while working as pressure sensors. PVDF and PVDF co-polymer nanofibers produced by electrospinning represent the most promising materials for flexible tactile-sensing applications.

## Figures and Tables

**Table 1:** The piezoelectric properties of PVDF, P(VDF-TrFE) and their nanocomposite via electrospinning.

Piezoelectric Material/Substrate	Output Voltage/Conductivity	Output Current	Sensitivity	Detection Limit	Stability (Cycles)	Ref.
PVDF nanofabrics	140 mV		42.00 mV/N			[28]
PVDF nanofibers webs	1–2.6 V	1.4–4.5 μA				[33]
PVDF nanofibers mats/configuration of PET and PDMS	100 mV at 0.025 MPa		5.812 mV kPa^−1^			[18]
aligned PVDF nanofibers mats	~3 mV					[88]
PVDF/MWCNT nanofiber webs	6 V		the volume conductivity is 5 orders higher than pure PVDF nanofibers			[94]
PVDF-0.05MWCNT-0.1OMMT	58 ± 2.5 mV		10.9 ± 1.25 mV/N			[93]
	48 ± 4.7 mV (pure PVDF)						
			8.84 ± 1.57 mV/N (pure PVDF)			
AgNWs doped PVDF nanofibers			29.8 pC/N (for *d*_33_)			[96]
			18.1 pC/N (pure PVDF)			
PVDF/PPy nanofibers	1.6 S·cm^−1^		40-fold increase in the relative conductivity			[99]
	3.2 × 10^−16^ S·cm^−1^ (pure PVDF)					
PVDF/PPy mats	10^7^ Ω·cm		200 Ω·cm/Pa	<0.02 MPa	>25	[98]
	10^17^ Ω·cm (pure PVDF)		20 Ω·cm/Pa (pure PVDF)			
P(VDF-TrFE) nanofibers layer/PI	0.5–1.5 V	6–40 nA		<0.1 Pa	1000	[34]
P(VDF-TrFE) nanofibers layer/PDMS	~2000 mV		120 mV/µm		>1000	[106]
P(VDF-TrFE) nanofibers	~5 mV		60.5 mV/N			[112]
P(VDF-TrFE) nanofibers	~0.7 V					[110]
P(VDF-TrFE) nanofiber webs			15.6 kPa^−1^	1.2 Pa	100,000	[114]
P(VDF-TrFE) nanofibers film/PDMS-MWCNT membrane	25 V (triboelectric voltage)	~6.5 μA (triboelectric current)				[111]
	2.5 V (piezoelectric voltage)	~2.3 μA (piezoelectric current)				
aligned P(VDF-TrFE) nanofibers	300 ± 5 mV					[115]
aligned P(VDF-TrFE) nanofibers			110.37 pC/Pa			[113]
P(VDF-TrFE) nanofibers (3D sensor)/PDMS	>1200 mV (flat shape)		23 VN^−1^ (flat shape)			[35]
	~1000 mV (wrist shape)		20 VN^−1^ (wrist shape)			
	~500 mV (finger shape)		12 VN^−1^ (finger shape)			
P(VDF-TrFE) (shell)-PVP/PEDOT: PSS (core) nanofibers	>1.6 V		4 mV/mmHg			[116]

## References

[1] Yang Y., Zhang H., Lin Z.-H., Zhou Y.S., Jing Q., Su Y., Yang J., Chen J., Hu C., Wang Z.L. (2013). Human skin based triboelectric nanogenerators for harvesting biomechanical energy and as self-powered active tactile sensor system. ACS Nano.

[2] Park M., Park Y.J., Chen X., Park Y.K., Kim M.S., Ahn J.H. (2016). MoS_2_-based tactile sensor for electronic skin applications. Adv. Mater..

[3] Lee M.H., Nicholls H.R. (1999). Review Article Tactile sensing for mechatronics—A state of the art survey. Mechatronics.

[4] Yang T., Xie D., Li Z., Zhu H. (2017). Recent advances in wearable tactile sensors: Materials, sensing mechanisms, and device performance. Mater. Sci. Eng. R Rep..

[5] Zhan C., Yu G., Lu Y., Wang L., Wujcik E., Wei S. (2017). Conductive polymer nanocomposites: A critical review of modern advanced devices. J. Mater. Chem. C.

[6] Kim S.Y., Park S., Park H.W., Park D.H., Jeong Y., Kim D.H. (2015). Highly Sensitive and Multimodal All-Carbon Skin Sensors Capable of Simultaneously Detecting Tactile and Biological Stimuli. Adv. Mater..

[7] Park S., Kim H., Vosgueritchian M., Cheon S., Kim H., Koo J.H., Kim T.R., Lee S., Schwartz G., Chang H. (2014). Stretchable Energy-Harvesting Tactile Electronic Skin Capable of Differentiating Multiple Mechanical Stimuli Modes. Adv. Mater..

[8] Barlian A.A., Park W.-T., Mallon J.R., Rastegar A.J., Pruitt B.L. (2009). Semiconductor piezoresistance for microsystems. Proc. IEEE.

[9] He R., Yang P. (2006). Giant piezoresistance effect in silicon nanowires. Nat. Nanotechnol..

[10] Zhou J., Gu Y., Fei P., Mai W., Gao Y., Yang R., Bao G., Wang Z.L. (2008). Flexible piezotronic strain sensor. Nano Lett..

[11] Gong S., Schwalb W., Wang Y., Chen Y., Tang Y., Si J., Shirinzadeh B., Cheng W. (2014). A wearable and highly sensitive pressure sensor with ultrathin gold nanowires. Nat. Commun..

[12] Lipomi D.J., Vosgueritchian M., Tee B.C., Hellstrom S.L., Lee J.A., Fox C.H., Bao Z. (2011). Skin-like pressure and strain sensors based on transparent elastic films of carbon nanotubes. Nat. Nanotechnol..

[13] Yao H.B., Ge J., Wang C.F., Wang X., Hu W., Zheng Z.J., Ni Y., Yu S.H. (2013). A flexible and highly pressure-sensitive graphene–polyurethane sponge based on fractured microstructure design. Adv. Mater..

[14] Kim K.K., Hong S., Cho H.M., Lee J., Suh Y.D., Ham J., Ko S.H. (2015). Highly sensitive and stretchable multidimensional strain sensor with prestrained anisotropic metal nanowire percolation networks. Nano Lett..

[15] Zhao S., Zhang G., Gao Y., Deng L., Li J., Sun R., Wong C.-P. (2014). Strain-driven and ultrasensitive resistive sensor/switch based on conductive alginate/nitrogen-doped carbon-nanotube-supported Ag hybrid aerogels with pyramid design. ACS Appl. Mat. Interfaces.

[16] Park J.W., Jang J. (2015). Fabrication of graphene/free-standing nanofibrillar PEDOT/P (VDF-HFP) hybrid device for wearable and sensitive electronic skin application. Carbon.

[17] Yan C., Wang J., Kang W., Cui M., Wang X., Foo C.Y., Chee K.J., Lee P.S. (2014). Highly stretchable piezoresistive graphene–nanocellulose nanopaper for strain sensors. Adv. Mater..

[18] Wu D., Huang S., Xiao Z., Yu L., Wang L., Sun D., Lin L. (2015). Poly (vinylidene fluoride) piezoelectric nanofibers fabricated by non-uniform field electrospinning. Int. J. Nanomanuf..

[19] Stassi S., Cauda V., Canavese G., Pirri C.F. (2014). Flexible tactile sensing based on piezoresistive composites: A review. Sensors.

[20] Pötschke P., Brünig H., Janke A., Fischer D., Jehnichen D. (2005). Orientation of multiwalled carbon nanotubes in composites with polycarbonate by melt spinning. Polymer.

[21] Pötschke P., Andres T., Villmow T., Pegel S., Brünig H., Kobashi K., Fischer D., Häussler L. (2010). Liquid sensing properties of fibres prepared by melt spinning from poly(lactic acid) containing multi-walled carbon nanotubes. Compos. Sci. Technol..

[22] Bautista-Quijano J.R., Pötschke P., Brünig H., Heinrich G. (2016). Strain sensing, electrical and mechanical properties of polycarbonate/multiwall carbon nanotube monofilament fibers fabricated by melt spinning. Polymer.

[23] Schwartz G., Tee B.C., Mei J., Appleton A.L., Kim D.H., Wang H., Bao Z. (2013). Flexible polymer transistors with high pressure sensitivity for application in electronic skin and health monitoring. Nat. Commun..

[24] Hwang E.-S., Seo J.-H., Kim Y.-J. (2007). A polymer-based flexible tactile sensor for both normal and shear load detections and its application for robotics. J. Microelectromechanical Syst..

[25] Dahiya R.S., Metta G., Valle M., Sandini G. (2010). Tactile sensing—From humans to humanoids. IEEE Trans. Rob..

[26] Chen S., Lou Z., Chen D., Chen Z., Jiang K., Shen G. (2016). Highly flexible strain sensor based on ZnO nanowires and P(VDF-TrFE) fibers for wearable electronic device. Sci. China Mater..

[27] Xin Y., Tian H., Guo C., Li X., Sun H., Wang P., Lin J., Wang S., Wang C. (2016). PVDF tactile sensors for detecting contact force and slip: A review. Ferroelectrics.

[28] Wang Y., Zheng J., Ren G., Zhang P., Xu C. (2011). A flexible piezoelectric force sensor based on PVDF fabrics. Smart Mater. Struct..

[29] Al-Saygh A., Ponnamma D., AlMaadeed M.A., Vijayan P.P., Karim A., Hassan M.K. (2017). Flexible Pressure Sensor Based on PVDF Nanocomposites Containing Reduced Graphene Oxide-Titania Hybrid Nanolayers. Polymers.

[30] An’amt M., Radiman S., Huang N., Yarmo M.A., Ariyanto N., Lim H., Muhamad M. (2010). Sol–gel hydrothermal synthesis of bismuth–TiO 2 nanocubes for dye-sensitized solar cell. Ceram. Int..

[31] Nabar B.P., Celik-Butler Z., Butler D.P. (2015). Self-powered tactile pressure sensors using ordered crystalline ZnO nanorods on flexible substrates toward robotic skin and garments. IEEE Sens. J..

[32] Huang Z.-M., Zhang Y.-Z., Kotaki M., Ramakrishna S. (2003). A review on polymer nanofibers by electrospinning and their applications in nanocomposites. Compos. Sci. Technol..

[33] Fang J., Niu H., Wang H., Wang X., Lin T. (2013). Enhanced mechanical energy harvesting using needleless electrospun poly (vinylidene fluoride) nanofibre webs. Energy Environ. Sci..

[34] Persano L., Dagdeviren C., Su Y., Zhang Y., Girardo S., Pisignano D., Huang Y., Rogers J.A. (2013). High performance piezoelectric devices based on aligned arrays of nanofibers of poly (vinylidenefluoride-co-trifluoroethylene). Nat. Commun..

[35] Han Bit L., Young Won K., Jonghun Y., Nak Kyu L., Suk-Hee P. (2017). 3D customized and flexible tactile sensor using a piezoelectric nanofiber mat and sandwich-molded elastomer sheets. Smart Mater. Struct..

[36] Li D., Xia Y. (2004). Electrospinning of nanofibers: Reinventing the wheel?. Adv. Mater..

[37] Agarwal S., Greiner A., Wendorff J.H. (2009). Electrospinning of manmade and biopolymer nanofibers—Progress in techniques, materials, and applications. Adv. Funct. Mater..

[38] Formhals A. (1934). Process and Apparatus for Preparing Artificial Threads. U.S. Patent.

[39] Teo W.E., Ramakrishna S. (2006). A review on electrospinning design and nanofibre assemblies. Nanotechnology.

[40] Persano L., Camposeo A., Tekmen C., Pisignano D. (2013). Industrial upscaling of electrospinning and applications of polymer nanofibers: A review. Macromol. Mater. Eng..

[41] Reneker D.H., Yarin A.L. (2008). Electrospinning jets and polymer nanofibers. Polymer.

[42] Luzio A., Canesi E.V., Bertarelli C., Caironi M. (2014). Electrospun polymer fibers for electronic applications. Materials.

[43] Yarin A.L., Koombhongse S., Reneker D.H. (2001). Taylor cone and jetting from liquid droplets in electrospinning of nanofibers. J. Appl. Phys..

[44] Reneker D.H., Yarin A.L., Fong H., Koombhongse S. (2000). Bending instability of electrically charged liquid jets of polymer solutions in electrospinning. J. Appl. Phys..

[45] Chang C., Limkrailassiri K., Lin L. (2008). Continuous near-field electrospinning for large area deposition of orderly nanofiber patterns. Appl. Phys. Lett..

[46] Sun D., Chang C., Li S., Lin L. (2006). Near-Field Electrospinning. Nano Lett..

[47] Huang Y., Bu N., Duan Y., Pan Y., Liu H., Yin Z., Xiong Y. (2013). Electrohydrodynamic direct-writing. Nanoscale.

[48] Zong X., Kim K., Fang D., Ran S., Hsiao B.S., Chu B. (2002). Structure and process relationship of electrospun bioabsorbable nanofiber membranes. Polymer.

[49] Doshi J., Reneker D.H. (1995). Electrospinning process and applications of electrospun fibers. J. Electrostat..

[50] Kuo C.C., Wang C.T., Chen W.C. (2008). Highly-Aligned Electrospun Luminescent Nanofibers Prepared from Polyfluorene/PMMA Blends: Fabrication, Morphology, Photophysical Properties and Sensory Applications. Macromol. Mater. Eng..

[51] Teo W.-E., Inai R., Ramakrishna S. (2011). Technological advances in electrospinning of nanofibers. Sci. Technol. Adv. Mater..

[52] Tuck S.J., Leach M.K., Feng Z.-Q., Corey J.M. (2012). Critical variables in the alignment of electrospun PLLA nanofibers. Mater. Sci. Eng. C.

[53] Yang D., Lu B., Zhao Y., Jiang X. (2007). Fabrication of aligned fibrous arrays by magnetic electrospinning. Adv. Mater..

[54] Wu Y., Carnell L.A., Clark R.L. (2007). Control of electrospun mat width through the use of parallel auxiliary electrodes. Polymer.

[55] Holmes-Siedle A., Wilson P., Verrall A. (1983). PVDF: An electronically-active polymer for industry. Mater. Des..

[56] Lovinger A.J. (1983). Ferroelectric polymers. Science.

[57] Nandi A., Mandelkern L. (1991). The influence of chain structure on the equilibrium melting temperature of poly (vinylidene fluoride). J. Polym. Sci. Part B: Polym. Phys..

[58] Salimi A., Yousefi A. (2003). Analysis method: FTIR studies of β-phase crystal formation in stretched PVDF films. Polym. Test..

[59] Giannetti E. (2001). Semi-crystalline fluorinated polymers. Polym. Int..

[60] Wu W., Wen X., Wang Z.L. (2013). Taxel-addressable matrix of vertical-nanowire piezotronic transistors for active and adaptive tactile imaging. Science.

[61] Correia H.M., Ramos M.M. (2005). Quantum modelling of poly (vinylidene fluoride). Comput. Mater. Sci..

[62] Pan H., Na B., Lv R., Li C., Zhu J., Yu Z. (2012). Polar phase formation in poly (vinylidene fluoride) induced by melt annealing. J. Polym. Sci. Part B: Polym. Phys..

[63] Sencadas V., Moreira M.V., Lanceros-Méndez S., Pouzada A.S., Gregório Filho R. (2006). α-to β Transformation on PVDF Films Obtained by Uniaxial Stretch.

[64] Ribeiro C., Sencadas V., Ribelles J.L.G., Lanceros-Méndez S. (2010). Influence of processing conditions on polymorphism and nanofiber morphology of electroactive poly (vinylidene fluoride) electrospun membranes. Soft Mater..

[65] Sencadas V., Gregorio R., Lanceros-Méndez S. (2009). α to β phase transformation and microestructural changes of PVDF films induced by uniaxial stretch. J. Macromol. Sci..

[66] Gradys A., Sajkiewicz P., Adamovsky S., Minakov A., Schick C. (2007). Crystallization of poly (vinylidene fluoride) during ultra-fast cooling. Thermochim. Acta.

[67] El Mohajir B.-E., Heymans N. (2001). Changes in structural and mechanical behaviour of PVDF with processing and thermomechanical treatments. 1. Change in structure. Polymer.

[68] Wu Y., Hsu S.L., Honeker C., Bravet D.J., Williams D.S. (2012). The role of surface charge of nucleation agents on the crystallization behavior of poly (vinylidene fluoride). J. Phys. Chem. B.

[69] Martins P., Caparros C., Gonçalves R., Martins P., Benelmekki M., Botelho G., Lanceros-Mendez S. (2012). Role of nanoparticle surface charge on the nucleation of the electroactive β-poly (vinylidene fluoride) nanocomposites for sensor and actuator applications. J. Phys. Chem. C.

[70] Lund A., Gustafsson C., Bertilsson H., Rychwalski R.W. (2011). Enhancement of β phase crystals formation with the use of nanofillers in PVDF films and fibres. Compos. Sci. Technol..

[71] Henkel K., Lazareva I., Mandal D., Paloumpa I., Müller K., Koval Y., Müller P., Schmeißer D. (2009). Electrical investigations on metal/ferroelectric/insulator/semiconductor structures using poly [vinylidene fluoride trifluoroethylene] as ferroelectric layer for organic nonvolatile memory applications. J. Vac. Sci. Technol. B: Microelectron. Nanometer Struct. Process. Measurement Phenom..

[72] He L., Sun J., Wang X., Wang C., Song R., Hao Y. (2013). Facile and effective promotion of β crystalline phase in poly (vinylidene fluoride) via the incorporation of imidazolium ionic liquids. Polym. Int..

[73] Eswaraiah V., Balasubramaniam K., Ramaprabhu S. (2012). One-pot synthesis of conducting graphene–polymer composites and their strain sensing application. Nanoscale.

[74] Wang D., Bao Y., Zha J.-W., Zhao J., Dang Z.-M., Hu G.-H. (2012). Improved dielectric properties of nanocomposites based on poly (vinylidene fluoride) and poly (vinyl alcohol)-functionalized graphene. ACS Appl. Mat. Interfaces.

[75] Liu Z., Pan C., Lin L., Lai H. (2013). Piezoelectric properties of PVDF/MWCNT nanofiber using near-field electrospinning. Sens. Actuators A.

[76] Glauß B., Steinmann W., Walter S., Beckers M., Seide G., Gries T., Roth G. (2013). Spinnability and characteristics of polyvinylidene fluoride (PVDF)-based bicomponent fibers with a carbon nanotube (CNT) modified polypropylene core for piezoelectric applications. Materials.

[77] Dror Y., Salalha W., Khalfin R.L., Cohen Y., Yarin A.L., Zussman E. (2003). Carbon nanotubes embedded in oriented polymer nanofibers by electrospinning. Langmuir.

[78] Ge J.J., Hou H., Li Q., Graham M.J., Greiner A., Reneker D.H., Harris F.W., Cheng S.Z. (2004). Assembly of well-aligned multiwalled carbon nanotubes in confined polyacrylonitrile environments: Electrospun composite nanofiber sheets. J. Am. Chem. Soc..

[79] Manna S., Nandi A.K. (2007). Piezoelectric β polymorph in poly (vinylidene fluoride)-functionalized multiwalled carbon nanotube nanocomposite films. J. Phys. Chem. C.

[80] Shah D., Maiti P., Gunn E., Schmidt D.F., Jiang D.D., Batt C.A., Giannelis E.P. (2004). Dramatic enhancements in toughness of polyvinylidene fluoride nanocomposites via nanoclay-directed crystal structure and morphology. Adv. Mater..

[81] Priya L., Jog J. (2002). Poly (vinylidene fluoride)/clay nanocomposites prepared by melt intercalation: Crystallization and dynamic mechanical behavior studies. J. Polym. Sci. Part B: Polym. Phys..

[82] Ahn Y., Lim J.Y., Hong S.M., Lee J., Ha J., Choi H.J., Seo Y. (2013). Enhanced piezoelectric properties of electrospun poly (vinylidene fluoride)/multiwalled carbon nanotube composites due to high β-phase formation in poly (vinylidene fluoride). J. Phys. Chem. C.

[83] Martins P., Lopes A., Lanceros-Mendez S. (2014). Electroactive phases of poly (vinylidene fluoride): Determination, processing and applications. Prog. Polym. Sci..

[84] Zheng J., He A., Li J., Han C.C. (2007). Polymorphism control of poly (vinylidene fluoride) through electrospinning. Macromol. Rapid Commun..

[85] Ueberschlag P. (2001). PVDF piezoelectric polymer. Sens. Rev..

[86] Wang Z.L. (2012). Progress in piezotronics and piezo-phototronics. Adv. Mater..

[87] Hosoda K., Tada Y., Asada M. (2006). Anthropomorphic robotic soft fingertip with randomly distributed receptors. Robot. Auton. Syst..

[88] Edmondson D., Cooper A., Jana S., Wood D., Zhang M. (2012). Centrifugal electrospinning of highly aligned polymer nanofibers over a large area. J. Mater. Chem..

[89] Yee W.A., Kotaki M., Liu Y., Lu X. (2007). Morphology, polymorphism behavior and molecular orientation of electrospun poly(vinylidene fluoride) fibers. Polymer.

[90] Laiarinandrasana L., Besson J., Lafarge M., Hochstetter G. (2009). Temperature dependent mechanical behaviour of PVDF: Experiments and numerical modelling. Int. J. Plast.

[91] Vinogradov A., Holloway F. (1999). Electro-mechanical properties of the piezoelectric polymer PVDF. Ferroelectrics.

[92] Li Z., Zhang X., Li G. (2014). In situ ZnO nanowire growth to promote the PVDF piezo phase and the ZnO–PVDF hybrid self-rectified nanogenerator as a touch sensor. PCCP.

[93] Hosseini S.M., Yousefi A.A. (2017). Piezoelectric sensor based on electrospun PVDF-MWCNT-Cloisite 30B hybrid nanocomposites. Org. Electron..

[94] Yu H., Huang T., Lu M., Mao M., Zhang Q., Wang H. (2013). Enhanced power output of an electrospun PVDF/MWCNTs-based nanogenerator by tuning its conductivity. Nanotechnology.

[95] Liu Y.-L., Li Y., Xu J.-T., Fan Z.-Q. (2010). Cooperative effect of electrospinning and nanoclay on formation of polar crystalline phases in poly (vinylidene fluoride). ACS Appl. Mat. Interfaces.

[96] Li B., Zheng J., Xu C. (2013). Silver Nanowire Dopant Enhancing Piezoelectricity of Electrospun PVDF Nanofiber Web. Proc. SPIE.

[97] Li B., Xu C., Zheng J., Xu C. (2014). Sensitivity of Pressure Sensors Enhanced by Doping Silver Nanowires. Sensors.

[98] Merlini C., Barra G., Araujo T.M., Pegoretti A. (2014). Electrically pressure sensitive poly (vinylidene fluoride)/polypyrrole electrospun mats. RSC Adv..

[99] Merlini C., dos Santos Almeida R., D’Ávila M.A., Schreiner W.H., de Oliveira Barra G.M. (2014). Development of a novel pressure sensing material based on polypyrrole-coated electrospun poly (vinylidene fluoride) fibers. Mater. Sci. Eng. B.

[100] Kakade M.V., Givens S., Gardner K., Lee K.H., Chase D.B., Rabolt J.F. (2007). Electric field induced orientation of polymer chains in macroscopically aligned electrospun polymer nanofibers. J. Am. Chem. Soc..

[101] Dias J.C., Correia D.M., Botelho G., Lanceros-Méndez S., Sencadas V. (2014). Electrical properties of intrinsically conductive core–shell polypyrrole/poly(vinylidene fluoride) electrospun fibers. Synth. Met..

[102] Costa C.M., Gomez Ribelles J.L., Lanceros-Méndez S., Appetecchi G.B., Scrosati B. (2014). Poly(vinylidene fluoride)-based, co-polymer separator electrolyte membranes for lithium-ion battery systems. J. Power Sources.

[103] Chu B., Zhou X., Ren K., Neese B., Lin M., Wang Q., Bauer F., Zhang Q. (2006). A dielectric polymer with high electric energy density and fast discharge speed. Science.

[104] Sun C., Shi J., Bayerl D.J., Wang X. (2011). PVDF microbelts for harvesting energy from respiration. Energy Environ. Sci..

[105] Baniasadi M., Huang J., Xu Z., Moreno S., Yang X., Chang J., Quevedo-Lopez M.A., Naraghi M., Minary-Jolandan M. (2015). High-performance coils and yarns of polymeric piezoelectric nanofibers. ACS Appl. Mat. Interfaces.

[106] Park S.-H., Lee H.B., Yeon S.M., Park J., Lee N.K. (2016). Flexible and stretchable piezoelectric sensor with thickness-tunable configuration of electrospun nanofiber mat and elastomeric substrates. ACS Appl. Mat. Interfaces.

[107] Nunes-Pereira J., Ribeiro S., Ribeiro C., Gombek C.J., Gama F., Gomes A., Patterson D., Lanceros-Méndez S. (2015). Poly (vinylidene fluoride) and copolymers as porous membranes for tissue engineering applications. Polym. Test..

[108] Xu H., Cheng Z.-Y., Olson D., Mai T., Zhang Q., Kavarnos G. (2001). Ferroelectric and electromechanical properties of poly (vinylidene-fluoride-trifluoroethylene-chlorotrifluoroethylene) terpolymer. Appl. Phys. Lett..

[109] Ma W., Yuan H., Wang X. (2014). The effect of chain structures on the crystallization behavior and membrane formation of poly (vinylidene fluoride) copolymers. Membranes.

[110] Mandal D., Yoon S., Kim K.J. (2011). Origin of Piezoelectricity in an Electrospun Poly (vinylidene fluoride-trifluoroethylene) Nanofiber Web-Based Nanogenerator and Nano-Pressure Sensor. Macromol. Rapid Commun..

[111] Wang X., Yang B., Liu J., Zhu Y., Yang C., He Q. (2016). A flexible triboelectric-piezoelectric hybrid nanogenerator based on P (VDF-TrFE) nanofibers and PDMS/MWCNT for wearable devices. Sci. Rep..

[112] Ren G., Cai F., Li B., Zheng J., Xu C. (2013). Flexible Pressure Sensor Based on a Poly (VDF-TrFE) Nanofiber Web. Macromol. Mater. Eng..

[113] Ke J.-Y., Chu H.-J., Hsu Y.-H., Lee C.-K. (2017). A Highly Flexible Piezoelectret-Fiber Pressure Sensor Based on Highly Aligned P (VDF-TrFE) Electrospun Fibers.

[114] Lou Z., Chen S., Wang L., Jiang K., Shen G. (2016). An ultra-sensitive and rapid response speed graphene pressure sensors for electronic skin and health monitoring. Nano Energy.

[115] Sharma T., Langevine J., Naik S., Aroom K., Gill B., Zhang J.X.J. Aligned electrospun PVDF-TrFE nanofibers for flexible pressure sensors on catheter. Proceedings of the 2013 Transducers & Eurosensors XXVII: The 17th International Conference on Solid-State Sensors, Actuators and Microsystems (TRANSDUCERS & EUROSENSORS XXVII).

[116] Sharma T., Naik S., Langevine J., Gill B., Zhang J.X. (2015). Aligned PVDF-TrFE nanofibers with high-density PVDF nanofibers and PVDF core-shell structures for endovascular pressure sensing. IEEE Trans. Biomed. Eng..

